# Circular RNA 103862 Promotes Proliferation and Invasion of Laryngeal Squamous Cell Carcinoma Cells Through the miR-493-5p/GOLM1 Axis

**DOI:** 10.3389/fonc.2020.01064

**Published:** 2020-07-29

**Authors:** Xin Wang, Tianyi Wu, Peng Wang, Like Yang, Qiuying Li, Jingting Wang, Rui Zhao, Jiarui Zhang, Ming Liu, Jing Cao, Linli Tian, Boyu Yu, Yanan Sun

**Affiliations:** ^1^Department of Otolaryngology, Head and Neck Surgery, The Second Affiliated Hospital, Harbin Medical University, Harbin, China; ^2^Department of Otorhinolaryngology, Head and Neck Surgery, Henan Provincial People's Hospital, Zhengzhou University People's Hospital, Henan University People's Hospital, Zhengzhou, China

**Keywords:** circular RNA, circRNA_103862, LSCC, miR-493-5p, GOLM1

## Abstract

Accumulating evidence suggests that circular RNAs (circRNAs) may be a key contributor to oncogenesis. Yet, the function of circRNAs in laryngeal squamous cell carcinoma (LSCC) is still not clear. In this study, we examined the function of circRNA_103862 in LSCC progression by analyzing the tissue specimens collected from a patient with LSCC by using different LSCC cell models *in vitro* and an LSCC xenograft model in nude mice. We found that circRNA_103862 was frequently upregulated in the tissues of LSCC and was correlated with metastasis and prognosis of LSCC patients. Furthermore, circRNA_103862 downregulation could reduce proliferation, migration, and invasion ability of LSCC cells. In terms of mechanism exploration, miR-493-5p was sponged by circRNA_103862. Rescue experiments also showed that circRNA_103862 could achieve a carcinogenic effect by regulating miR-493-5p. Moreover, a luciferase reporter analysis showed that Golgi membrane protein 1 (GOLM1) is a downstream effector of miR-493-5p. In conclusion, our data suggested that circRNA_103862 promotes the proliferation of LSCC through targeting the miR-493-5p/GOLM1 axis, and it might serve as a potential prognosis marker and therapy target for LSCC.

## Introduction

Laryngeal squamous cell carcinoma (LSCC) is a common tumor of head and neck (1). Primary surgery, systemic chemotherapy, and local radiotherapy are the most common treatment approaches for LSCC. Despite the improvement in treatment strategy, the survival rate of LSCC patients remains low (2, 3). A recent meta-analysis indicated that a 5-year overall survival rate of LSCC patients is lower than 64.3% (4). Another study revealed a 5-year overall survival for the T3N+ stage of LSCC of 33% (5). Therefore, more advanced and effective treatments and diagnostic methods for LSCC are urgently needed.

Circular RNAs (circRNAs), a new type of non-coding RNAs widely expressed in mammals, are characterized by covalently closed-loop structures with neither 5′ to 3′ polarity nor polyadenylated tail (6, 7). The circRNAs have been involved in a variety of cell functions, including sponging small RNA, binding to RNA-binding proteins, and protein translation process (8–11). Furthermore, it has been recently discovered that those single-stranded RNAs have an essential role in the development of colorectal cancer, hepatocellular carcinoma, and other cancers (12, 13). Moreover, some studies showed that circRNAs might act as a biomarker in the diagnosis or prognosis of multiple cancers due to its tissue-specific expression pattern and significant stability.

In our previous study, we examined the role of circRNA (circRNA_103862) in LSCC, which was identified through a high-throughput Arraystar Human circRNA Microarray (14). CircRNA_103862 (named has_circ_0072758, circBase, http://www.circbase.org/) was the second upregulated circular RNA in LSCC (fold change = 81.328, *P* < 0.001). In order to evaluate the feasibility of circRNA_103862 as a diagnostic biomarker and therapeutic target, we first examined its presence in LSCC tissues. We found that the circRNA_103862 was upregulated in LSCC tissues and was correlated with the survival rate of LSCC patients. Moreover, we discovered that downregulation of circRNA_103862 could inhibit LSCC cell proliferation, migration, and invasion *in vitro*.

Golgi membrane protein 1 (GOLM1) has been identified as a novel regulator in a variety of cancers, yet its role in LSCC remains unclear. Our data suggested that GOLM1 expression is increased and correlated with the prognosis of LSCC. In this study, we discovered that circRNA_103862 might regulate GOLM1 by sponging miR-493-5p in LSCC. Our findings provide new insights into the regulatory mechanisms of circRNA_103862 in LSCC progression.

## Materials and Methods

### Tissue Specimens

A total of 62 paired human LSCC and adjacent non-tumor tissues were collected between 2017 and 2018 from the Department of Otorhinolaryngology of the Second Affiliated Hospital of Harbin Medical University. LSCC was confirmed by pathology. In addition, patients with LSCC had no history of radiotherapy and chemotherapy. Patients with a second primary tumor, distant metastasis, or histopathological diagnosis other than squamous cell carcinoma were excluded. Furthermore, paraffin sections of 152 patients with LSCC-paired normal tissues were collected from the Department of Pathology of the Second Affiliated Hospital of Harbin Medical University for *in situ* hybridization (ISH) and immunohistochemistry (IHC) examination. This study was approved by the Ethical Committee of Harbin Medical University, and all patients provided written informed consent (ethical approval number: KY2017-047).

### *In situ* Hybridization

The expression of circRNA_103862 in LSCC tissues was examined by ISH employing the RNAscope technology (Brown, USA). circRNA_103862 target probe, along with the positive and negative control probes, were provided by Advanced Cell Diagnostics (ACD). Briefly, after collection, tissues were fixed in formalin and prepared for ISH (*n* = 152) analysis based on the manufacturer's instructions. The cytoplasm or nucleus was stained with brown dots, indicating positive results. The slides were rated at 200× magnification according to the RNAscope grading guide; the five grades are shown in Table 1. The scores 0 and 1 were identified as low expression, and scores 2–4 were identified as high expression of circRNA_103862.

**Table 1:** RNAscope grading guide.

**CircRNA_103862**	**Score**	**Scoring guide**
Low expression	0	1–3 spots/tumor cell
	1	4–10 spots/tumor cell
High expression	2	>10 spots/tumor cell
	3	>10 spots/tumor cell and <10% of the tumor cells had clusters of spots
	4	>10 spots/tumor cell and >10% of the tumor cells had clusters of spots

### RT-qPCR

The experimental method referred to this article (14). Relative quantification of circRNA_103862 expression was calculated by 2^−ΔΔ*Ct*^ method. The primer sequences were listed as follows: circRNA_103862, forward: 5′-GATACTGCCTCTCCAAGCCCAATG-3′, reverse: 5′-GGTCTGACTGCTTGCTCTTCCTC-3′; GOLM1, forward: 5′-GTGCTGGTGCCAGCCTGTTA-3′, reverse: 5′-AGTGCTCTAGGCCATTGATTGATTG-3′; miR-493-5p, forward: 5′-TCCTACGGAGAGGCTCAG-3′, reverse: 5′-TCCTCGTAGTCCAACACG-3′; glyceraldehyde 3-phosphate dehydrogenase (GAPDH), forward: 5′-TCGACAGTCAGCCGCATCTTCTTT-3′, reverse: 5′-ACCAAATCCGTTGACTCCGACCTT-3′.

### Cell Transfection

AMC-HN-8 cell line (LSCC cell) was sourced from Shanghai Yu Bo Biotechnology Co., Ltd. (Shanghai, China), TU 212 cell line (LSCC cell) was sourced from Shanghai Biowing applied Biotechnology Co., Ltd. (Shanghai, China), and 16HBE human bronchial epithelial cell line (ATCC® CRL-2741^TM^) was provided by American Type Culture Collection (ATCC; Manassas, VA, USA). The method and condition of cell culture were described in a previous article (13). Genechem (Shanghai, China) designed and synthesized small-interfering RNA (siRNA) targeting circRNA_103862 (si-circ) and siRNA negative control (si-NC); miR-493-5p mimic, mimic negative control (miR-NC), and miR-493-5p inhibitor (inh-miR-493-5p); circRNA_103862 overexpression vector (over-circ) and empty vector (vector). The transfection protocol was performed according to the manufacturer's instruction.

### Cell Proliferation and Colony Formation

The Cell Counting Kit-8 (CCK-8) assay (Dojindo Laboratories, Japan) was used to analyze cell proliferation. A total of 5,000 transfected AMC-HN-8 and TU212 cells/well were seeded in the 96-well plates and maintained for 48 h; this procedure was performed in triplicate. At each time point (0, 24, 48, 72, and 96 h), 10 μl CCK-8 reagent was added to each well, and the plate was incubated for an additional 4 h. A microplate reader (Bio-Rad, Richmond, CA, USA) was used to measure the absorbance at 450 nm.

For colony formation experiment, 500 transfected AMC-HN-8 and TU212 cells/well were seeded into six-well plates for ~12 days. After the clones were formed, cells were fixed with paraformaldehyde and stained with crystal violet.

### Cell Migration

Wound healing assay was used to assess the cell migration, which was carried out according to a previously described approach (15).

### Cell Invasion

The matrix gel invasion chamber was used to measure the invasion ability of cells. Briefly, 1 × 10^5^ cells were incubated in serum-free medium and inoculated into the upper chamber of each insert (24-well plate, 8-μm aperture, Corning), while the lower chamber contained culture medium containing 20% fetal bovine serum. After 48 h, the cotton swab was used to remove the remaining cells from the upper surface, while the cells invading the upper surface were fixed with 4% paraformaldehyde and stained with 5% crystal violet. Subsequently, five fields were randomly selected under the microscope to observe and count the number of invasive cells.

### Establishing Xenografts in Mice

Twenty BALB/c nude mice, 6 weeks old, were provided by Beijing Vital River Laboratory Animal Technology Co. Ltd. All the animals were housed according to a previously described approach (15). Mice were randomly divided into four groups (*n* = 5/group): experimental group 1, which was subcutaneously injected with circRNA_103862 downregulated TU212 cells in the dorsal region; experimental group 2, which was subcutaneously injected with circRNA_103862 overexpression AMC-HN-8 cells in the dorsal region; two control groups that were injected with the same amount of TU212 or AMC-HN-8, respectively. The size of the tumor was measured twice a week with calipers, and the volume of xenografts was determined using the formula of a rotational ellipsoid (length × width^2^ × 0.5). Mice were euthanized 3 weeks after cell injection, and the tumor was collected and further analyzed. LSCC cell proliferation was examined using a proliferating cell nuclear antigen (PCNA) in LSCC xenografts.

### Luciferase Reporter Assays

LSCC cells with circRNA_103862 reporter (wild or mutant type) or empty reporter vector, cotransfected with miR-493-5p mimics or mimics control, were cultured in 24-well plate. Twenty-four hours later, a dual-luciferase reporter system (Genechem Shanghai, China) was used to detect luciferase activity. The specific activity of the target was measured by the relative activity ratio of firefly luciferase to Renilla luciferase. In order to test whether miR-493-5p-targeted GOLM1, WT GOLM1 UTR reporter plasmid (GOLM1-WT) and mutant GOLM1-3′UTR reporter plasmid (GOLM1-mut) were, respectively, constructed by pmirGLA promoter vector.

### Immunohistochemistry Assay

LSCC and xenografts fixed specimen with ethanol dehydration grading series solution were implanted in paraffin and cut into 4-μm sections. Samples were then dewaxed and rehydrated with xylene and ethanol. The antigen was extracted with high-pressure heat. Consequently, samples were blocked with normal sheep serum at 37°C for 10 min, primary antibody GOLM1 (1:400, ab109628), and PCNA (1:200, sc-56) were used to incubate the samples at 4°C all night, after which they were washed with phosphate-buffered saline (PBS). Finally, all slices were dehydrated, removed, fixed, and displayed by colorimetry based on diaminophenylhydrazine. The cells containing the brown particles were positive cells, and the expression of GOLM1 and PCNA was calculated by the positive cells/all cells ratio: high expression of >25% (+) and low expression of <25% (–).

### Western Blotting

Protease inhibitor cocktail was added to radioimmunoprecipitation assay (RIPA) lysate (Beyotime, China); LSCC cells are lysed with this mixed solution. After separating the protein samples by sodium dodecyl sulfate–polyacrylamide gel electrophoresis (SDS-PAGE), they were transferred to the polyvinylidene fluoride (PVDF) membrane, which was sealed with Blocking Buffer (Beyotime), and detected with primary anti-GOLM1 (Santa Cruz, CA, USA). GAPDH was used as an internal control of the same membrane. Horseradish peroxidase (HRP) was used as the secondary antibody to incubate the membrane for 1 h, and the immune response signal was visualized by the ECL detection system (Beyotime).

### Data Analysis

SPSS 17.0 statistical software package was employed for all statistical analyses. Two samples were compared and analyzed by Student's *t*-test. *X*^2^ test was used to evaluate the correlation between circRNA_103862 and clinicopathological features. Kaplan–Meier survival curve was used to calculate the overall survival of LSCC patients. The diagnostic value was evaluated by receiver operating characteristic (ROC) curve. *P* < 0.05 was considered to be statistically significant.

## Results

### circRNA_103862 Is Highly Expressed in LSCC Tissues and Cell Lines

**Figure 1:**
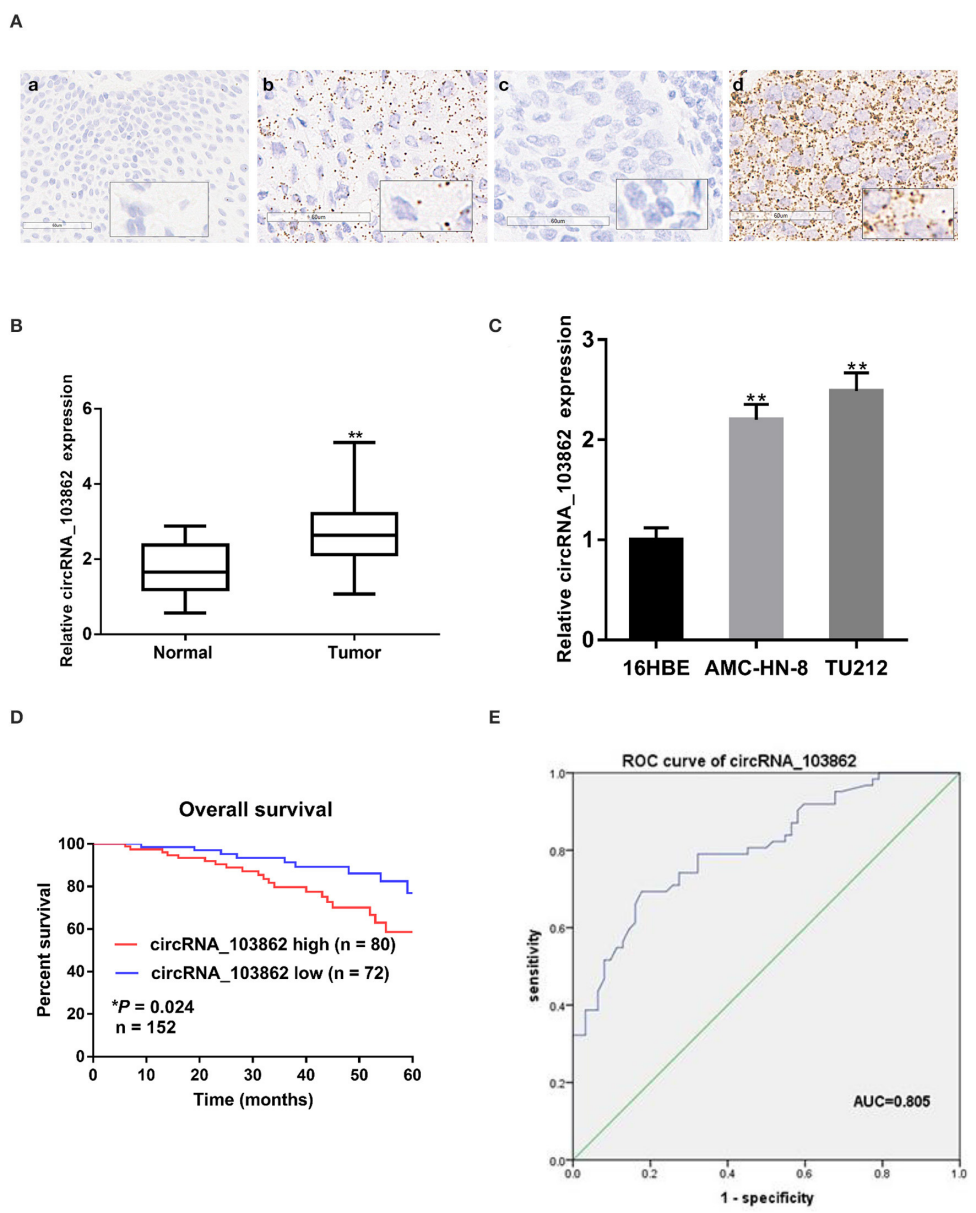
Relative expression of circRNA_103862 in laryngeal squamous cell carcinoma (LSCC) tissues and cell lines and its clinical significance. **(A)** Representative *in situ* hybridization (ISH) images of circRNA_103862 expression in LSCC. (a) Adjacent tissue; (b) LSCC tissue (positive results are indicated with an arrow); (c) negative control; (d) positive control. **(B)** The Boxplot shows the circRNA_103862 expression in 62 paired LSCC cancer and paracancerous tissues (Student's *t*-test). **(C)** Relative expression circRNA_103862 of in LSCC cell lines and normal cell lines measured by reverse transcription quantitative PCR (RT-qPCR). **(D)** The correlation between survival time and circRNA_103862 expression was analyzed in LSCC patients, *P = 0.024. **(E)** Receiver operating characteristic (ROC) curve analysis to evaluate the diagnostic value of circRNA_103862. **(E)** The area under the curve (AUC) was 0.805 (95% CI, 0.729–0.880, *P = 0.039). *P < 0.05, **P < 0.01.

ISH results indicated that the level of circRNA_103862 in 152 LSCC tissues was higher than that in the corresponding adjacent tissues. In addition, circRNA_103862 was mainly located in the cytoplasm (Figure 1A). Consistently, the expression of circRNA_103862 was analyzed by reverse transcription quantitative PCR (RT-qPCR) in LSCC tissues (*n* = 62) and cell lines. The results displayed that the level of circRNA_103862 in LSCC tissues was higher than that in non-cancerous tissues (*P* < 0.01, Figure 1B). Moreover, in AMC-HN-8 and TU212 cells, the circRNA_103862 was significantly upregulated compared to the normal cell line 16HBE (Figure 1C).

### circRNA_103862 Is Associated With the Progression and Survival of LSCC

According to the ISH expression of circRNA_103862 in LSCC tissue samples, the patients with LSCC were divided into high-expression group (*n* = 80) and low-expression group (*n* = 72). Fisher's exact test showed that a higher level of circRNA_103862 was closely related to the clinical stage (*P* = 0.026) and lymph-node metastasis (*P* = 0.019). At the same time, circRNA_103862 expression was not correlated with sex, age, smoking history, and T stage (*P* > 0.05 for all, Table 2). Moreover, the Kaplan–Meier survival curve showed that the increase in circRNA_103862 level in LSCC tissues was significantly related to the poor prognosis of LSCC patients (log-rank test; chi square = 5.0387; *P* = 0.024, Figure 1D). In addition, the area under the receiver operating characteristic (ROC) curve of circRNA_103862 in discriminating 62 LSCC tissues and 62 normal tissues was 0.805. As shown in Figure 1E, circRNA_103862 was proved to have diagnostic value (sensitivity = 0.823, specificity = 0.694, Youden's index = 0.517, *P* < 0.001). To sum up, these data indicate that circRNA_103862 may be a potential biomarker for LSCC patients' diagnosis and prognosis.

**Table 2:** Correlation between circRNA_103862 expression and clinicopathological parameters in laryngeal squamous cell carcinoma (LSCC) patients.

**Characteristics**	**circRNA_103862 expression**		***P-*value^**a**^**
	**High (*n* = 80)**	**Low (*n* = 72)**	
Age (years)			0.065
≥60 (96)	56	40	
<60 (56)	24	32	
Gender			0.359
Male (131)	67	64	
Female (21)	13	8	
Smoking status			0.190
Non-smoker (122)	61	61	
Smoker (30)	19	11	
Clinical stage			0.026^*^
I/II (87)	39	48	
III/IV (65)	41	24	
T stage			0.752
T1/2 (93)	48	45	
T3/4 (59)	32	27	
Nodal metastasis			0.019^*^
Positive (57)	37	20	
Negative (95)	43	52	

* *P < 0.05*.

a *chi-square test*.

### circRNA_103862 Knockdown Inhibits the Proliferation of LSCC Cells *in vitro* and *in vivo*

**Figure 2:**
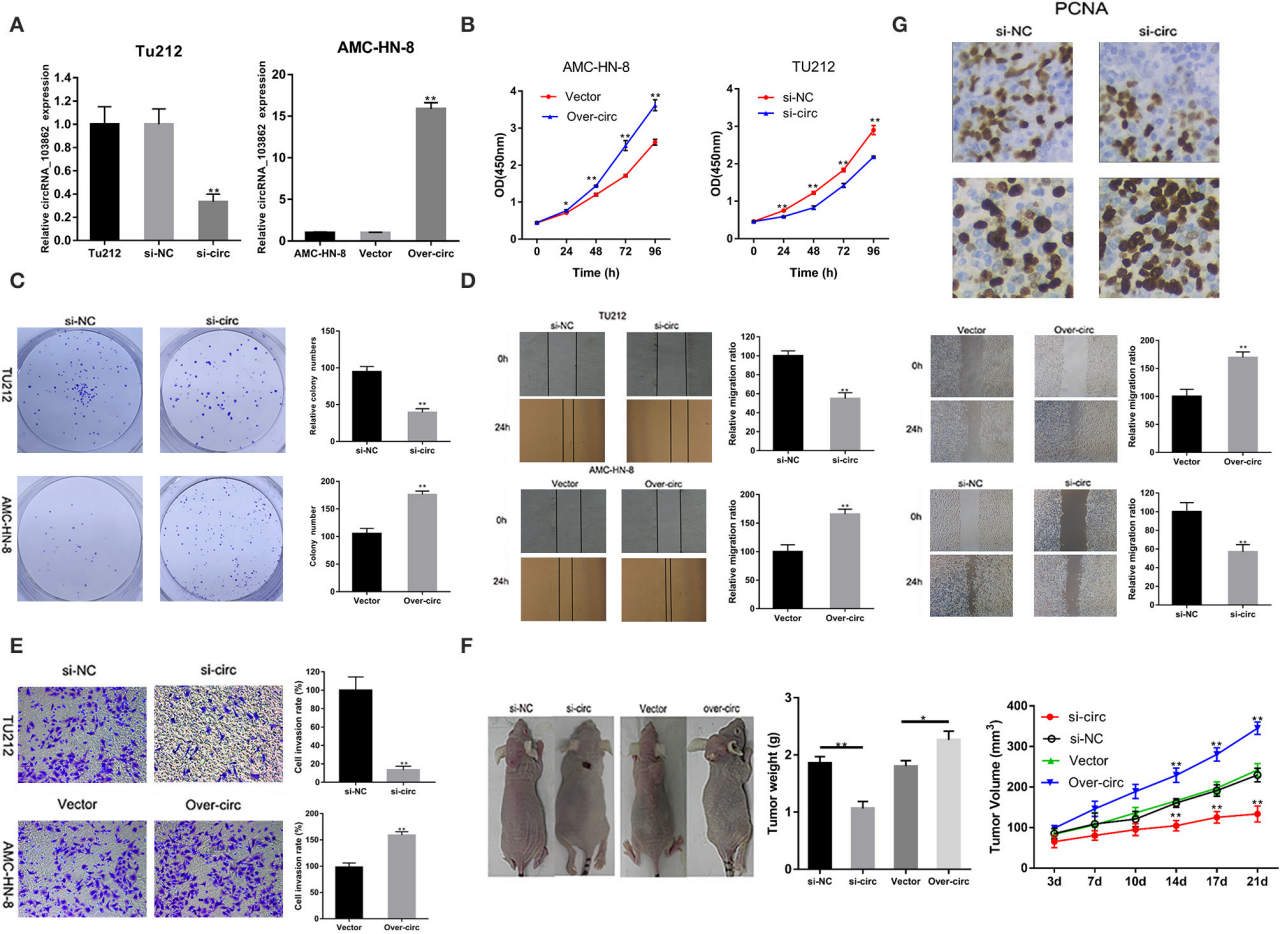
CircRNA_103862 promotes laryngeal squamous cell carcinoma (LSCC) cell progression. **(A)** CircRNA_103862 expression was detected after transfection in TU-212 cells by reverse transcription quantitative PCR (RT-qPCR); circRNA_103862 expression was detected after transfection in AMC-HN-8 cells by RT-qPCR. **(B)** Cell Counting Kit-8 (CCK-8) assays were used to analyze the cell viability of TU-212 and AMC-HN-8 cells after transfection. **(C)** Colony formation assays were used to detect the clone ability of TU-212 and AMC-HN-8 cells after transfection. **(D)** Wound healing assays were used to analyze the migration ability of TU-212 and AMC-HN-8 cells after transfection. **(E)** Transwell assays were used to detect cell invasion capacities of TU-212 and AMC-HN-8 cells after transfection. **(F)** The downregulated expression of circRNA_103862 inhibited the growth of LSCC *in vivo*. **(G)** The PCNA assay was performed by immunohistochemistry. *P < 0.05, **P < 0.01 (**A–G** vs. control or as indicated by Student's *t*-test).

The high expression of circRNA_103862 in LSCC tissues indicated its tumorigenic effect in LSCC cells. In order to evaluate the si-circRNA_103862 function in TU212 cells, the knockout efficiency was verified by RT-qPCR; the overexpression efficiency of circRNA_103862 in AMC-HN-8 cell line was also determined (Figure 2A). As shown in Figures 2B,C, circRNA_103862 knockout significantly reduced the cell viability and cloning ability of TU212 cell lines, while its upregulation caused the opposite effect. Similarly, the effects of circRNA_103862 on migratory and invasive potentials of TU212 and AMC-HN-8 cells were reversed by circRNA_103862 interference as well (Figures 2D,E).

Furthermore, we evaluated the biological behavior of tumor cells *in vivo*, using a xenograft animal model. These data were consistent with the *in vitro* results. The tumor growth in mice injected with circRNA_103862-knockdown TU212 cells was significantly inhibited compared with mice injected with AMC-HN-8 cells overexpressing circRNA_103862 (Figure 2F). To further evaluate the proliferation of LSCC cells *in vivo*, the immunohistochemistry assay was used to analyze the expression of PCNA in mouse xenografts. Results showed that the expression of PCNA was lower in mouse xenografts with circRNA_103862 downregulation compared to those with circRNA_103862 overexpression (Figure 2G).

### circRNA_103862 Acts as a Sponge for miR-493-5p

**Figure 3:**
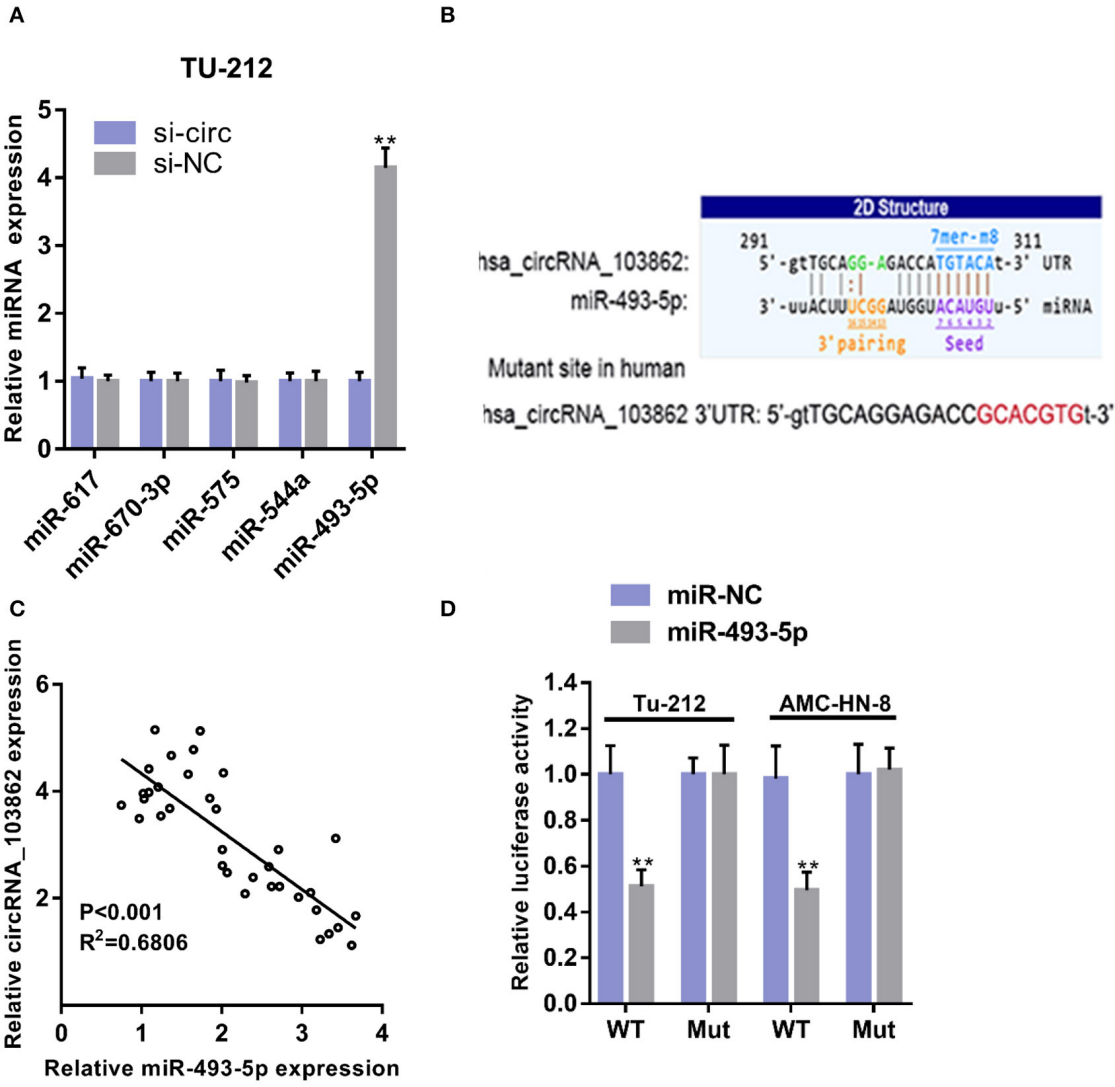
CircRNA_103862 sponges miR-493-5p. **(A)** Relative microRNA (miRNA) expression was detected after transfection in TU-212 cells (Student's *t*-test). **(B)** Diagrammatic sketch of the binding sites for circRNA_103862 and miR-493-5p. **(C)** Spearman's correlation analysis of circRNA_103862 and miR-493-5p in LSCC patients' tissues. **(D)** Luciferase reporter assay was conducted to evaluate the interaction ability between miR-493-5p and circRNA_103862 (Student's *t*-test). **P < 0.01.

**Figure 4:**
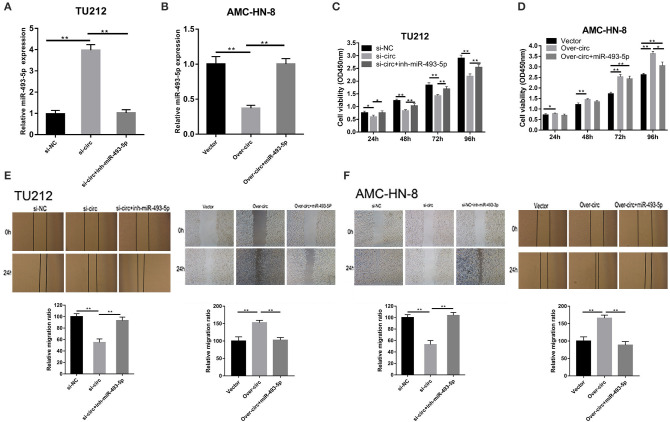
The oncogenic role of circRNA_103862 is partly dependent on its regulation of miR-493-5p. **(A,B)** Reverse transcription quantitative PCR (RT-qPCR) was used to detect miR-493-5p expression after transfection in TU212/ AMC-HN-8 cells. **(C,D)** Cell Counting Kit-8 (CCK-8) assays were used to evaluate cell viability after transfection in TU212/ AMC-HN-8 cells. **(E,F)** Wound healing assays were used to evaluate TU212/AMC-HN-8 cells migration ability after transfection in cells. *P < 0.05, **P < 0.01 (**A–F** vs. control or as indicated by Student's *t*-test).

Using starBase (http://starbase.sysu.edu.cn/), we found the microRNAs (miRNAs) (miR-617, miR-670-3p, miR-575, miR-493-5p, and miR-544a) that could potentially be sponged by circRNA_103862. The data of RT-qPCR showed that miR-493-5p were negatively regulated by circRNA_103862 (Figure 3A). The seeding sequences of circRNA_103862 and miR-493-5p are shown in Figure 3B. Besides, the correlation analysis showed that circRNA_103862 was negatively correlated with miR-493-5p (Figure 3C). To verify whether miR-493-5p was a direct target of circRNA_103862, the AMC-HN-8 and TU212 cells were cotransfected with indicated mimics and wild-type (WT) or mutated-type (Mut) circRNA_103862-luciferase reporter. Therefore, miR-493-5p mimics significantly inhibited the luciferase activity mediated by WT circRNA_103862-luciferase reporter, implying an interaction between circRNA_103862 and miR-493-5p (Figure 3D). After verifying the interaction between circRNA_103862 and miR-493-5p, we further examined whether the carcinogenic function caused by circRNA_103862 was related to its inhibition of miR-493-5p. We found that cotransfection with si-circRNA_103862 and miR-493-5p inhibitor could significantly reverse the upregulation of miR-493-5p induced by si-circRNA_103862 (Figure 4A). In addition, cotransfection with miR-493-5p mimics restored the downregulation of miR-493-5p caused by circRNA_103862 vectors (Figure 4B).

Subsequently, we evaluated the potential regulatory effects of circRNA_103862 and miR-493-5p in LSCC using CCK8 and wound healing experiments. Our results showed that miR-493-5p inhibitors could partially rescue the cancer-inhibiting function of si-circRNA_103862. In addition, the silencing of miR-493-5p almost completely restored the carcinogenic function of the TU212 cell line (Figure 4C). miR-493-5p partially mimicked the reversed carcinogenic effect caused by the overexpression of circRNA_103862. In the over-circ+miR-493-5p group, the cell proliferative ability of the AMC-HN-8 cell line was further weakened (Figure 4D). The scratch test results were consistent with CCK8 (Figures 4E,F).

### circRNA_103862 Promotes LSCC Growth Through the miR-493-5p/GOLM1 Pathway

**Figure 5:**
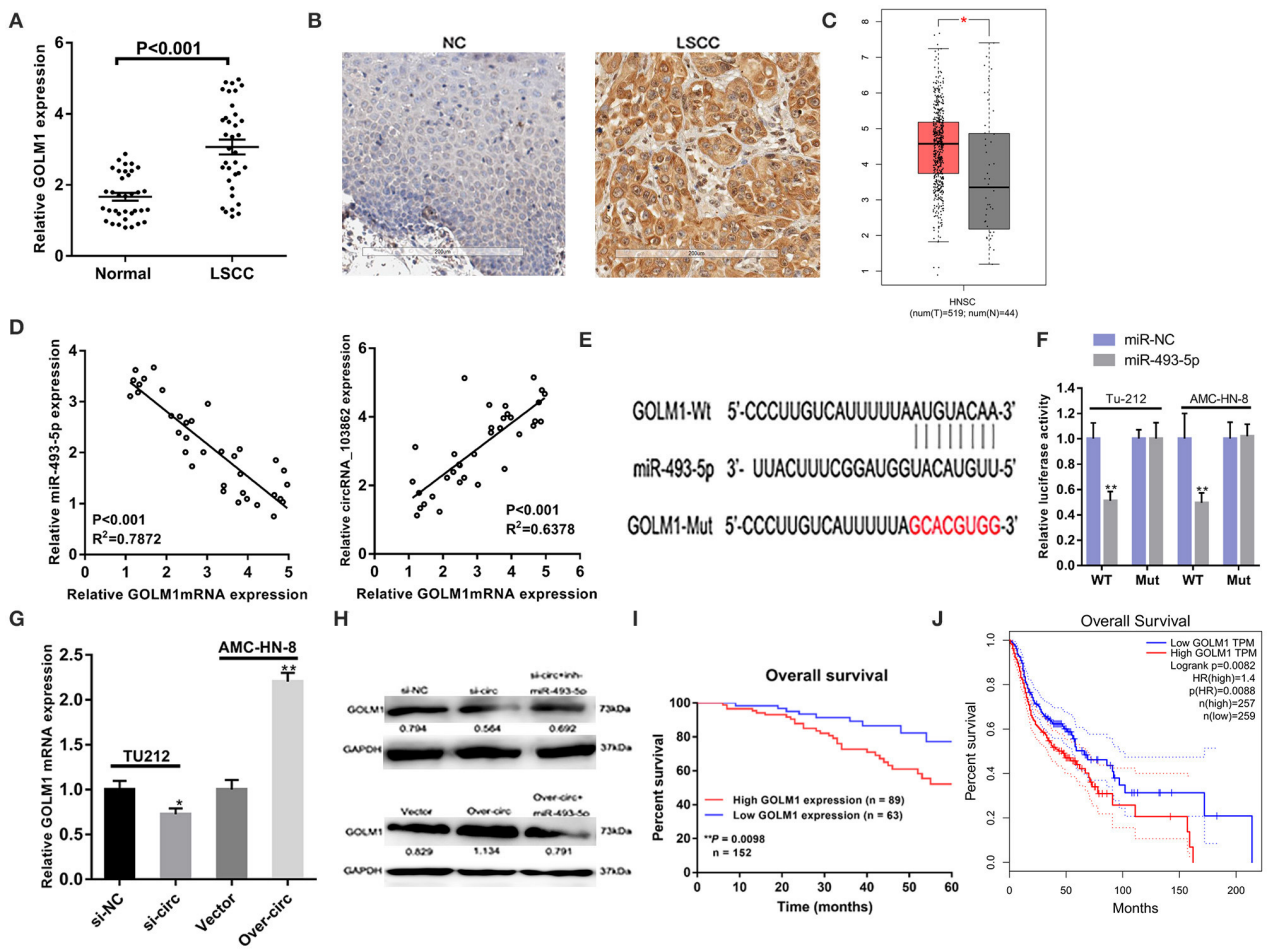
CircRNA_103862 promotes the growth of laryngeal squamous cell carcinoma (LSCC) through the circRNA_103862/miR-493-5p/GOLM1 pathway. **(A,B)** Both reverse transcription quantitative PCR (RT-qPCR) and immunohistochemistry (IHC) showed Golgi membrane protein 1 (GOLM1) was significantly upregulated in LSCC tissues compared with matched non-tumorous tissues (**A** Student's *t*-test; *B* positive results are indicated with an arrow). **(C)** GOLM1 expression in head and neck squamous cell carcinoma (HNSCC) and normal samples from the Gene Expression Profiling Interactive Analysis (GEPIA) HNSCC data set [match The Cancer Genome Atlas (TCGA) normal and Genotype-Tissue Expression (GTEx) data]. **(D)** GOLM1 was negatively correlated with miR-493-5p and positively correlated with circRNA_103862 in LSCC tissues (Spearman's correlation analysis). **(E,F)** The putative binding sites between GOLM1 and miR-493-5p were predicted, respectively, and the relative luciferase activity was expressed as fir/Renilla luciferase activity (**F** Student's *t*-test). **(G)** RT-qPCR was used to detect GOLM1 messenger RNA (mRNA) expression after transfection in TU212 and AMC-HN-8 cells (Student's *t*-test). **(H)** Protein expression of GOLM1 was inhibited after knockdown of circRNA_103862 and was regulated by inh-miR-493-5p. After circRNA_103862 overexpression, GOLM1 protein expression was regulated by miR-493-5p. **(I)** The correlation between survival time and GOLM1 expression was analyzed in LSCC patients. **(J)** Kaplan–Meier analyses of the correlations between GOLM1 expression and disease survival of 516 patients from the GEPIA HNSCC data set. The log-rank test was used to calculate *P*-values. *P < 0.05, **P < 0.01.

Bioinformatical analyses were further performed to predict the putative target genes of miR-493-5p. TargetScan and miRDB software predicated that GOLM1 might serve as a potential target of miR-493-5p. The binding sites of miR-493-5p and GOLM1 were predicted using microRNA.org (http://mirdb.org/). Through RT-qPCR and IHC, we found that the expression levels of GOLM1 messenger RNA (mRNA) and protein in LSCC tumor tissues were significantly higher than those in matched non-tumor tissues (Figures 5A,B). Besides, we analyzed the head and neck squamous cell carcinoma (HNSCC), which included LSCC from the Gene Expression Profiling Interactive Analysis (GEPIA) database (tumor group, 519; normal group, 44). GEPIA is a web-based tool, which can deliver fast and customizable functionalities based on The Cancer Genome Atlas (TCGA) and Genotype-Tissue Expression (GTEx) data. GEPIA is available at http://gepia.cancer-pku.cn/.

We found an upregulation of GOLM1 mRNA levels in HNSCC tissues (Figure 5C). Furthermore, the correlation analysis showed that GOLM1 was negatively correlated with miR-495-5p and positively correlated with circRNA_103862 (Figure 5D). The seeding sequences of miR-493-5p and GOLM1 are shown in Figure 5E. Luciferase reporter suggested the interaction between miR-493-5p and GOLM1 (Figure 5F). The expression levels of GOLM1 in LSCC cells were detected. RT-qPCR results showed that GOLM1 was significantly inhibited after circRNA_103862 downregulation. On the contrary, circRNA_103862 overexpression significantly increased the expression levels of GOLM1 (Figure 5G). The results of a Western blot also showed that GOLM1 expression was significantly inhibited after circRNA_103862 was silenced. The miR-493-5p inhibitor can partially rescue GOLM1 expression, which inhibits the function of si-circRNA_103862 (Figure 5H). Kaplan–Meier survival curve showed that the increase in the GOLM1 level in LSCC tissues was significantly related to the poor prognosis of LSCC patients (log-rank test; chi-square = 6.677; *P* = 0.0098, Figure 5I). Bioinformatics analysis of GEPIA HNSCC data showed that the highly expressed of GOLM1 was significantly correlated with disease-free survival in HNSCC tissues (log-rank test, *P* = 0.0088, Figure 5J).

## Discussion

Laryngeal cancer is considered to be one of the most important causes of cancer-related death in the world (16). Therefore, identifying new molecular targets is a key step in improving the diagnosis, prognosis, and treatment of LSCC.

CircRNA is considered a novel biomarker and therapeutic target for human cancer. Numerous studies have shown that circRNAs have an important role in human cancers (17–19). Circ-RNAs function as miRNA sponge (20); it may interact with proteins (21) and regulate protein translation and gene transcription (22). Among them, the theory of competing for endogenous RNA (ceRNA) has been extensively studied and accepted. According to this theory, some circRNAs, such as hsa_circ_0000092/miR-338-3p in hepatocellular carcinoma (23), hsa_circ_0088732/miR-661 in glioma (24), circRNA-AKT1/miR-942-5p in cervical cancer (25), as well as circRASSF2/miR-302b-3p (26), and circ-CCND1/miR-646 in LSCC (27) can act as ceRNAs, interacting with miRNAs, thus reducing its negative regulatory effect on specific mRNA (28).

In this study, circRNA_103862 was significantly upregulated in the tissues and cells of LSCC, while its downregulation reduced the LSCC cell proliferation *in vitro* and *in vivo*. Moreover, higher expression of circRNA_103862 was associated with advanced clinical stage, lymphatic metastasis, and overall survival time in patients with LSCC. To further examine the biological function of circRNA_103862, we analyzed its ceRNA mechanism in LSCC. We identified the interaction between circRNA_103862 and miR-493-5p; this specific microRNA has an anticancer role in a variety of malignancies (29, 30). Zhang et al. (29) revealed that miR-493-5p exerts a tumor-suppressive role in spinal osteosarcoma and osteosarcoma cells. Moreover, Zhao et al. (30) suggested that miR-493-5p is a novel regulator of invasiveness and tumorigenicity of breast cancer cells through targeting FUT4. The miR-493-5p/FUT4 pathway has therapeutic potential in breast cancer. In this study, rescue experiments verified that the carcinogenic effect of circRNA_103862 depends on its inhibition of miR-493-5p.

GOLM1, also known as GOLPH2 and GP73, is a type II transmembrane protein of the Golgi cisternae, which is highly expressed in tumor cells and is regarded as a potential cancer cell marker (31, 32). GOLM1 has a carcinogenic role in a variety of cancers. For example, Zhang et al. (33) found that the expression of GOLM1 was upregulated in non-small cell lung cancer (NSCLC) and promoted its proliferation and invasion. Nevertheless, the role of GOLM1 in LSCC still remains unclear. Bioinformatics analysis showed that GOLM1 was highly expressed in HNSCC and was correlated with short survival time. Consistently, we found that GOLM1 is upregulated in LSCC tissues and that high level of GOLM1 was associated with poor prognosis. These results suggested that GOLM1 has an oncogenic function in LSCC. Moreover, we discovered that miR-493-5p could directly target the GOLM1 in LSCC cells, and the restoration of miR-493-5p could partially block circRNA_103862 carcinogenic role in LSCC by reducing the expression of GOLM1. In short, the upregulation of circRNA_103862 predicted dismal prognosis in LSCC and facilitated cell progression through the miR-493-5p/GOLM1 axis. Although more evidence is needed, our work revealed the relevant role of GOLM1 and therapeutic targets in LSCC.

In summary, we mainly investigated the function and mechanism of circRNA_103862 in LSCC. We discovered that circRNA_103862 is highly expressed in LSCC tissues. In addition, we found that circRNA_103862 overexpression promotes the proliferation of LSCC cells through competing for endogenous RNA regulatory mechanisms of miR-493-5p/GOLM1 axis. These findings suggest that circRNA_103862 could be used as a biomarker for its prognosis and a potential therapeutic target for LSCC. Although this study confirmed the carcinogenic effect of circRNA_103862 on LSCC, it was not possible to exclude that other circRNAs were involved in the carcinogenesis and progression of LSCC. Follow-up studies should add the RNA-seq or proteomics studies in LSCC cells with circRNA_103862 downregulation and overexpression to reveal the deeper mechanisms of circRNA_103862. Other potential targets of miR-493-5p in LSCC should be further studied, too.

## Data Availability Statement

All datasets generated for this study are included in the article/supplementary material.

## Ethics Statement

The studies involving human participants were reviewed and approved by the Ethical Committee of Harbin Medical University. The patients/participants provided their written informed consent to participate in this study. The animal study was reviewed and approved by the Ethical Committee of Harbin Medical University.

## Author Contributions

YS and BY designed the experiment. XW, TW, PW, BY, LY, RZ, JZ, ML, and JC made the experiment and analysis. LT, QL, and JW prepared the manuscript. All authors contributed to the article and approved the submitted version.

## Conflict of Interest

The authors declare that the research was conducted in the absence of any commercial or financial relationships that could be construed as a potential conflict of interest.
